# Electroacupuncture Ameliorates Learning and Memory and Improves Synaptic Plasticity via Activation of the PKA/CREB Signaling Pathway in Cerebral Hypoperfusion

**DOI:** 10.1155/2016/7893710

**Published:** 2016-10-18

**Authors:** Cai-Xia Zheng, Min Lu, Ya-Bi Guo, Feng-Xia Zhang, Hua Liu, Feng Guo, Xiao-Lin Huang, Xiao-Hua Han

**Affiliations:** ^1^Department of Rehabilitation Medicine, Tongji Hospital, Tongji Medical College, Huazhong University of Science and Technology, Wuhan, Hubei Province, China; ^2^Department of Rehabilitation Medicine, Xiangyang Central Hospital, Hubei College of Arts and Science, Xiangyang, Hubei Province, China; ^3^College of Health Science, Wuhan Institute of Physical Education, Wuhan, Hubei Province, China

## Abstract

Electroacupuncture (EA) has shown protective effects on cognitive decline. However, the underlying molecular mechanisms are ill-understood. The present study was undertaken to determine whether the cognitive function was ameliorated in cerebral hypoperfusion rats following EA and to investigate the role of PKA/CREB pathway. We used a rat 2-vessel occlusion (2VO) model and delivered EA at Baihui (GV20) and Dazhui (GV14) acupoints. Morris water maze (MWM) task, electrophysiological recording, Golgi silver stain, Nissl stain, Western blot, and real-time PCR were employed. EA significantly (1) ameliorated the spatial learning and memory deficits, (2) alleviated long-term potentiation (LTP) impairment and the reduction of dendritic spine density, (3) suppressed the decline of phospho-CREB (pCREB) protein, brain-derived neurotrophic factor (BDNF) protein, and microRNA132 (miR132), and (4) reduced the increase of p250GAP protein of 2VO rats. These changes were partially blocked by a selective protein kinase A (PKA) inhibitor, N-[2-(p-bromocinnamylamino)ethyl]-5-isoquinoline-sulfonamide (H89), suggesting that the PKA/CREB pathway is potentially involved in the effects of EA. Moreover, any significant damage to the pyramidal cell layer of CA1 subregion was absent. These results demonstrated that EA could ameliorate learning and memory deficits and alleviate hippocampal synaptic plasticity impairment of cerebral hypoperfusion rats, potentially mediated by PKA/CREB signaling pathway.

## 1. Introduction

Due to an increase in aging population, age-related cognitive impairment, in particular vascular cognitive impairment (VCI), becomes increasingly challenging worldwide, without effective medications [1]. As a novel combinational approach, electroacupuncture (EA) therapy, consisting of traditional acupuncture and modern electrotherapy technology, was frequently reported to alleviate cognitive decline in patients with stroke [2], Alzheimer disease (AD) [3, 4], or mild cognitive impairment (MCI) [5–7]. In addition, EA was suggested to prevent cognitive deficits in rats with cerebral ischemia [8–11]. However, the underlying molecular mechanisms are not entirely understood.

cAMP response element-binding protein (CREB)/CRE-mediated gene transcription and protein synthesis have been speculated to be essential in long-term hippocampal synaptic plasticity and memory formation [12–16]. Several pieces of evidence have confirmed that protein kinase A (PKA) is one of the major kinases to activate CREB and the PKA/CREB pathway is critical in learning and memory [17]. Previous studies had revealed that transcription of BDNF gene was CREB-regulated in an activity-dependent manner, wherein its expression was involved in neuronal development, synaptic plasticity, and neuroprotection [13]. For example, BDNF was related to the selective vulnerability of hippocampal neuronal populations during brain injury [18]. It also played a vital role in long-term potentiation (LTP), a cellular model for learning and memory [19, 20]. Of note, recently, miRNA132 (miR132) was identified as a novel CREB target gene and was suggested to regulate neuronal morphogenesis through translational inhibition of a GTPase-activating protein, p250GAP [21].

Hitherto, only a few studies about the molecular alterations of PKA/CREB pathway during EA treatment have been reported. In agreement, acupuncture treatment at Zusanli (ST36) was shown to increase cAMP concentration, PKA activity, pCREB protein, and pERK protein expressions, accompanied by alleviation of memory impairment in cerebral multi-infarction rats [8]. In addition, one of our previous studies had shown that EA attenuated neuronal apoptosis and ameliorated learning and memory in 2VO rats, via increasing the expressions of pCREB and BCL-2 protein [22].

Based on the above findings, we hypothesized that treatment of EA might enhance hippocampal synaptic plasticity and improve cognitive function of cerebral ischemic rats and that PKA/CREB signaling pathway might be a potential mechanism underlying these effects. Firstly, spatial learning and memory, LTP and dendritic spine density, and neuronal viability as well as expression of pCREB, BDNF, miR132, and p250GAP proteins were examined to test effects of EA in cerebral hypoperfusion. Furthermore, to test the hypothesis that the PKA/CREB pathway is involved in those beneficial effects of EA, rats were pregiven a selective PKA inhibitor, H89 (it can strongly inhibit the biological action of PKA), and similarly monitored for the above-mentioned indicators. Thus, the present study was aimed at determining whether and how the cognitive function and the hippocampal synaptic plasticity were altered by EA in cerebral hypoperfusion. Also, we investigated the probable underlying molecular machinery comprised of the PKA/CREB pathway.

## 2. Materials and Methods

### 2.1. Animals

A total of 87 adult male Sprague-Dawley rats weighing between 250 g and 300 g were used (SPF grade, Experimental Animal Center, Huazhong University of Science and Technology, Wuhan, China). Animals were housed in a group of 4-5/cage at 24 ± 1°C in a light-controlled (12 h light/dark cycle) room, providing free access to water and food. All the animal studies were approved by the Review Committee for the Care and Use of Laboratory Animals of Tongji Medical College, Huazhong University of Science and Technology.

### 2.2. Establishment of 2VO Model

Animals were randomly assigned into five groups: (1) control group: rats were anesthetized and exposed to surgery but did not deal with actual ligation; (2) model group: rats have undergone 2VO operation; (3) EA group: rats were given EA treatment after 2VO operation, for seven consecutive days; (4) EA+H89 group: rats were given H89 at 30 min before the operation of 2VO and then received EA similarly to the EA group; and (5) EA+normal saline (NS) solution group: rats were injected with equal volume of NS instead of H89, at 30 min before the 2VO operation and the subsequent EA treatment.

H89 (2 *μ*g/*μ*L × 10 *μ*L, Sigma-Aldrich, Shanghai, China) or a vehicle of sterile NS was intracerebroventricularly (icv) injected into rats of EA+H89 group or EA+NS group. Under intraperitoneal anesthesia (10% chloral hydrate, 350 mg/kg, intraperitoneally (ip)), the head was mounted in a stereotaxic instrument (SN-3, Narishige, Japan). Injection coordinates were 0.8 mm posteriorly to the bregma, 1.5 mm laterally to the midline in the right hemisphere, and 4.5 mm below the dural surface.

Through a ventral midline cervical incision, both common carotid arteries were carefully isolated from their sheaths and vagal nerves and doubly ligated with 4-0 silk thread. After occlusion, most animals appeared normal, but few rats that displayed epileptic seizures (2/87) or absence of weight gain (3/87) or intestinal obstruction (2/87) were excluded from subsequent experiments.

### 2.3. EA Treatment

**Figure 1:**
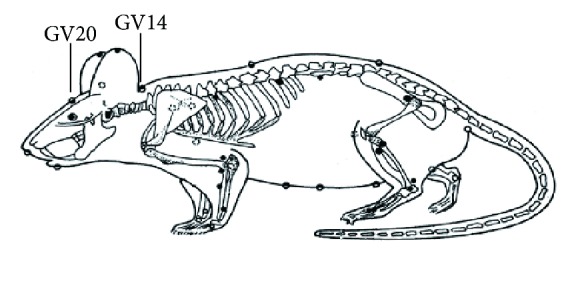
Rat schematic showing the location of the acupuncture points used in the study. GV14 represents “Dazhui,” which is located on the posterior midline and in the depression below the spinous process of the seventh cervical vertebra; GV20 represents “Baihui,” which is located at the right midpoint of the parietal bone.

EA was commenced one day after the operation in conscious rats. The location of “Baihui” acupoint (GV20) and “Dazhui” acupoint (GV14) was described previously [23] (Figure 1).

*“Acupuncture needles were inserted into the muscle at a depth of 0.5 mm of GV20 to serve as a cathode while another electrode was placed on the GV14 serving as an anode. Then electrical stimulation was delivered using a G6805-II electroacupuncture therapeutic apparatus (Shanghai Medical Electronic Apparatus Co., China), with continuous current at 20 Hz for 20 min daily. Stimulus intensity was based on the visible light facial muscles twitching.”*

### 2.4. Morris Water Maze Task

Morris water maze (MWM) task was carried out to test spatial learning and memory. Briefly, a circular water tank (150 cm in diameter and 50 cm deep) was filled with 23 ± 2°C water to a depth of 21 cm. A circular platform of 15 cm in diameter and 20 cm height was placed in the center of the target quadrant (quadrant I). Several visual cues were located on the wall of the test room. Rats were subjected to two sessions of four place navigation trials per day with an interval of at least 4 h for three consecutive days (day 5–day 7, training for 5 times). The starting points were changed for every trial. On day 8, the platform was removed for spatial probe trial to test the spatial memory. The latency to find the submerged platform and the dwell time in quadrant I and the swimming paths were recorded automatically using a computer-based image analyzer MWM tracking system MT-200 (ChengDu Technology & Market Co., Ltd., Chengdu, Sichuan Province, China).

### 2.5. Electrophysiological Recording

Rats were anesthetized with urethane (1.5 g/kg, ip) and placed in a stereotaxic frame (SN-3, Narishige, Japan) for surgery and recording. A bipolar stimulating electrode was placed into Schaffer collateral pathway of the dorsal hippocampus (4.5 mm posteriorly to bregma, 3.7 mm laterally to the midline, and at 2.5 mm depth from the brain surface). A recording electrode was positioned in the ipsilateral stratum radiatum underneath the CA1 area (3.5 mm posteriorly to bregma, 2.5 mm laterally, and at 2.0 mm depth from the brain surface). A 30 min rest period was implemented after the electrodes insertion.

Baseline responses were collected by low-frequency stimulation (0.05 Hz, width 0.1 ms, and 50% of maximal response) and recorded for 20 min. Subsequently, four 100 Hz trains of high-frequency stimulation (HFS, 80% of maximum response) were delivered, including 20 pulses and 30 s of intertrain intervals, lasting for an additional 20 min. Following HFS, field excitatory postsynaptic potentials (fEPSPs) were recorded, being set at 5 Hz at the same stimulus intensity as the baseline pulse, over 60 min. fEPSP slope level was calculated by the ratio of the absolute fEPSP slope to baseline value. It was defined that LTP was successfully induced if the fEPSP slope level was ≥120%. Evoked field responses were acquired, amplified, monitored, and analyzed with RM6240BD biology signal processing system (Chengdu Instrument Factory, China).

### 2.6. Golgi Silver Stain

Under anesthesia (10% chloral hydrate, 350 mg/kg, ip), rats underwent transcardial perfusion with 0.9% saline solution (containing 0.5% sodium nitrite), followed by 4% paraformaldehyde (PFA) in 0.1 M phosphate buffer (pH 7.4) for 2 h. Then the rats were perfused with a mordant dye comprising a mixture of 5% chloral hydrate, 5% potassium dichromate, 4% formaldehyde, and distilled water for another 1-2 h in dark until toes of the perfused rats turned to be heavy tangerine. Brains were taken out and coronally sectioned to three pieces, of which the middle ones were soaked in the mordant agent for 3 days. Consequently, the specimens were immersed in 1.5% silver nitrate solution for another 3 days. Finally, the brain sections were cut into 50 *μ*m slices on a vibratome (Campden Instrument, MA752, Leicester, UK) and rinsed with 2% potassium dichromate solution, dehydrated, cleared, and coverslipped.

Using Olympus BX51 microscope, images of dendrites of pyramidal neurons in the hippocampal CA1 region were traced for at least 50 *μ*m from secondary and tertiary dendritic branches at 1000x magnification. The density of dendritic spines along the length was estimated by spine number/10 *μ*m.

### 2.7. Histology

Under anesthesia, rats were perfused with 0.9% saline solution followed by 4% PFA in 0.1 M phosphate buffer, pH 7.4. Brains were then removed and fixed for 6–8 h at 4°C. Following paraffin embedding, from each rat were collected three coronal slices containing the dorsal hippocampus at 5 *μ*m thickness. Toluidine blue staining (Nissl staining) was performed. Representative photomicrographs of pyramidal cell layer of CA1 region were microscopically captured. Quantitative analysis of the ratios of viable neurons was processed by Image-Pro Plus (Leica DMLB) software at 400x magnification. An average of three Nissl-stained sections was calculated to yield the single parameter per rat. All microscopic analyses were conducted by an observer blinded to the groups.

### 2.8. Western Blot

Rats in each group were sacrificed by decapitation. Hippocampus tissues were quickly removed from the brain and homogenized in ice-cold lysate buffer containing 50 mmol/L Tris-HCl (pH = 8.0), 150 mmol/L NaCl, 1% Triton X-100, 100 *μ*g/mL PMSF, and phosphatase inhibitors (Sigma, USA). Proteins were fractionated on 10% SDS-PAGE gels and then transferred to polyvinylidene difluoride (PVDF) membrane (Merck Millipore, Germany). The blots were then incubated with phospho-CREB (Ser133) rabbit anti-rat monoclonal antibody (mAb) (1 : 1000, Cell Signaling Technology, Danvers, MA, USA), rabbit anti-rat polyclonal antibody for BDNF (1 : 300, Boster, Wuhan, China), goat anti-rat polyclonal antibody for p250GAP (1 : 200, Santa Cruz Biotechnology, Santa Cruz, CA, USA), or *β*-tubulin mouse anti-rat mAb (1 : 1000, Affinity Bioscience, USA) antibodies overnight at 4°C. The immunocomplexes were visualized with horseradish peroxidase (HRP) conjugated mouse anti-rabbit, mouse anti-goat, or rabbit anti-mouse secondary antibodies (1 : 5000, Proteintech Group Inc., Wuhan, China) by using an enhanced chemiluminescence (ECL) system (Millipore, Billerica, MA, US). Optical density of the bands was scanned and quantified with NIH ImageJ software and the results were normalized to the quantity of *β*-tubulin in each sample lane. All assays were repeated at least three times.

### 2.9. Quantitative Real-Time PCR

Hippocampal tissues were processed with RNAlater® Solution (Ambion® RNA by Life Technologies™, New York, USA) to stabilize and protect RNA before storage in liquid Nitrogen. Total RNA was extracted using TRIzol reagent (Invitrogen, Carlsbad, CA, USA). 10 ng RNA was reverse-transcribed into cDNA with the Toyobo First-Strand cDNA Synthesis Kit (USA). U6 RNA served as an endogenous reference. The reactions were carried out in triplicate in a StepOne™ Real-Time PCR System (Life Technologies, New York, USA) in a 10 *μ*L reaction mixture. The relative change of miR132 expression was determined using 2^−ΔΔCT^ method [24]. The primers for pre-miR132 were as follows: forward primer: 5′-ACCGTGGCTTTCGATTGTTAC-3′ and reverse primer: 5′-TGGTGTCGTGGAGTCG-3′, and those for U6 were as follows: forward primer: 5′-CCTGCTTCGGCAGCACA-3′ and reverse primer: 5′-AACGCTTCACGAATTTGCGT-3′.

### 2.10. Statistical Analysis

All data were presented as mean ± SEM and statistically analyzed with SPSS 19.0 software (IBM Corporation, Somers, New York, USA). The escape latencies in MWM test were tested by two-way ANOVA with repeated measures. The other data were analyzed by one-way ANOVA followed by post hoc test for multiple comparisons among the control group, model group, and EA group and were evaluated by independent-samples Student's *t*-test between the EA+H89 group and EA+NS group. *P* < 0.05 was considered as statistically significant.

## 3. Results

### 3.1. EA Ameliorated Spatial Learning and Memory Deficits Induced by 2VO, and the Effect Was Partially Inhibited by H89

**Figure 2:**
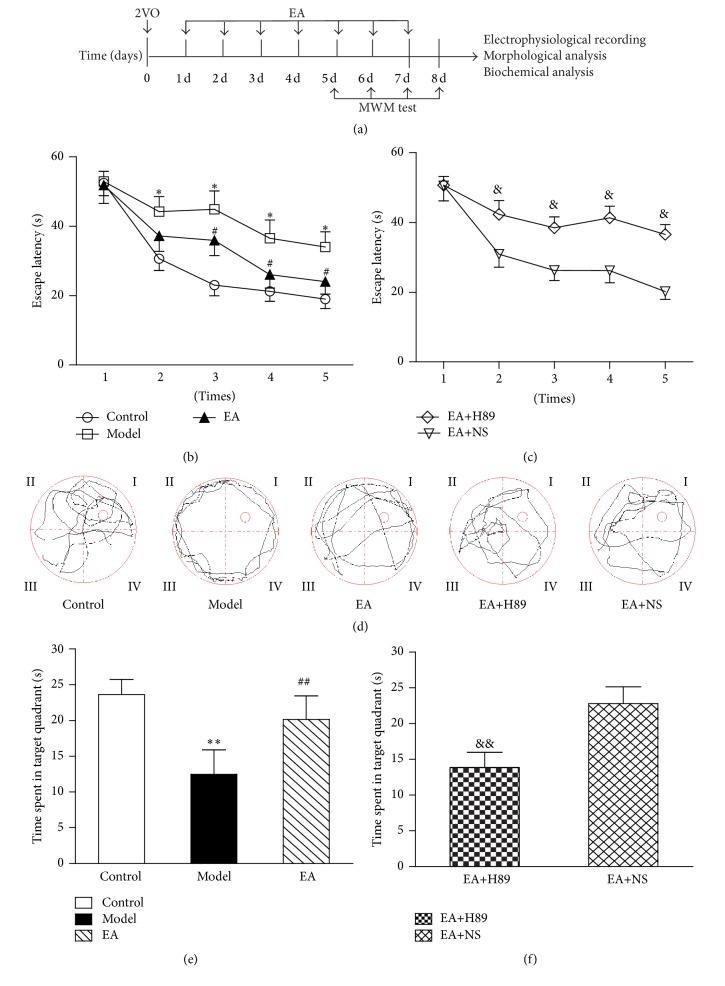
EA prevented spatial learning and memory deficits induced by 2VO, and the effect was partially inhibited by H89. (a) Timeline of the experiments to show the EA therapy and detection. (b and c) Escape latency to find the hidden platform from the 1st to the 5th time. Rats in the model group had longer escape latencies than the control group (b). EA could reverse the spatial learning impairment induced by 2VO. Rats in the EA+H89 group had longer escape latency than the EA+NS group rats (c). H89 could partly reverse the beneficial effect of EA. (d) The typical swimming paths of each group in the spatial probe trial. (e and f) Time spent in the target quadrant in the probe test. The amount of time expended in the target quadrant was significantly shorter in the model group rats than in the control group rats, and rats treated with EA exhibited significantly more time in the target quadrant (e). Rats in the EA+H89 group spent a shorter time in the target quadrant compared to EA+NS group rats (f). Each value represents mean ± SEM, *n* = 10 for each group. ^*∗∗*^ *P* < 0.01, compared with the control group; ^*∗*^ *P* < 0.05, compared with the control group; ^##^ *P* < 0.01, compared with the model group; ^#^ *P* < 0.05, compared with the model group; ^&&^ *P* < 0.01, compared with the EA+NS group; and ^&^ *P* < 0.05, compared with the EA+NS group.

The potential protective effects of EA against spatial learning and memory deficits of 2VO rats were assessed using the MWM test. The analysis of escape latencies showed significant differences between the control group, model group, and EA group (group effects: *F*(2,65) = 19.489, *P* = 0.000; training day effects: *F*(4,260) = 13.239, *P* = 0.000; and *n* = 10 per group). Compared with the control group, the model group rats exhibited significantly longer escape latencies from the 2nd to the 5th time during the training period (*P* < 0.05, resp., Figure 2(b)). Compared with the model group, the EA group rats shortened the escape latencies from the 3rd to the 5th time during the training period (*P* < 0.05, resp., Figure 2(b)). The analysis of escape latencies showed significant differences between the EA+H89 group and EA+NS group (group effects: *F*(1,38) = 18.913, *P* = 0.000; training day effects: *F*(4,152) = 12.944, *P* = 0.000; and *n* = 10 per group). The EA+H89 group rats demonstrated significantly prolonged escape latencies than the EA+NS group rats from the 2nd to the 5th time during the training period (*P* < 0.05, resp., Figure 2(c)). Typical swimming paths in the spatial probe trial of each group were displayed in Figure 2(d). Analyses with one-way ANOVA revealed a significant difference between the control group, model group, and EA group (*F*(2,27) = 5.470, *P* = 0.010; *n* = 10 per group). The time spent in the target quadrant was significantly decreased in the model group (*P* < 0.01 versus control group, Figure 2(e)), while it was significantly increased in the EA group (*P* < 0.01 versus model group, Figure 2(e)). The EA+H89 group rats exhibited significantly shorter time than that of the EA+NS group (*t*(9) = −2.902, *P* < 0.01 versus EA+NS group; *n* = 10 per group, Figure 2(f)).

### 3.2. EA Alleviated LTP Impairment and the Reduction of Dendritic Spine Density of 2VO Rats, and the Effect Was Partially Inhibited by H89

**Figure 3:**
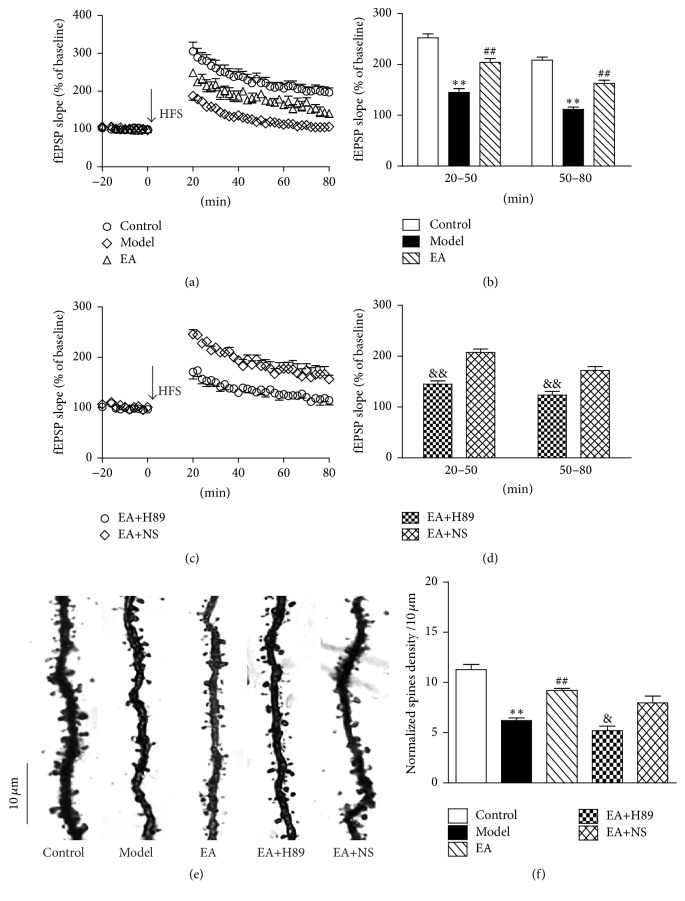
EA alleviated LTP impairment and the reduction of dendritic spine density of 2VO rats, and the effects were partially inhibited by H89. (a and c) The linear graph of the normalized fEPSP slope. The downward filled arrow indicates HFS. (b and d) The histogram of average fEPSP slope at 20–50 min and 50–80 min after HFS. The normalized fEPSP slope was significantly decreased in the model group. EA reversed the 2VO-induced LTP impairment, and this effect was partly inhibited by H89. Each value represents mean ± SEM, *n* = 10 for each group. (e) Representative dendritic segments of CA1 pyramidal neurons in the hippocampus (1000x, scale bar = 10 *μ*m). (f) Normalized mean dendrite spine density counts. EA attenuates 2VO-induced dendritic spine loss of CA1 region in the hippocampus, and this effect was partly inhibited by H89. Each value represents mean ± SEM, *n* = 6 for each group. ^*∗∗*^ *P* < 0.01, compared with the control group; ^##^ *P* < 0.01, compared with the model group; ^&&^ *P* < 0.01, compared with the EA+NS group; and ^&^ *P* < 0.05, compared with the EA+NS group.

The analysis of fEPSP slopes showed a significant difference among the control group, model group, and EA group (*F*(2,90) = 112.541, *P* = 0.000; *n* = 10 per group). As shown in Figures 3(a) and 3(b), HFS of the Schaffer collateral inputs to CA1 pyramidal cells induced a stable LTP in the slope of fEPSP in control rats (20–50 min after HFS: 252.22 ± 7.98% of baseline values; 50–80 min after HFS: 208.46 ± 5.91% of baseline values). Contrastingly, in the model group, fEPSP slope was significantly reduced (20–50 min after HFS: 144.88 ± 7.46% of baseline values; 50–80 min after HFS: 111.37 ± 4.66% of baseline values, *P* < 0.01 versus the control group). EA reversed the 2VO-induced LTP impairment (20–50 min after HFS: 204.19 ± 7.58% of baseline values; 50–80 min after HFS: 162.45 ± 6.37% of baseline values, *P* < 0.01 versus the model group). The normalized fEPSP slope was significantly decreased in EA+H89 group (20–50 min after HFS: 144.92 ± 6.50% of baseline values; 50–80 min after HFS: 123.52 ± 7.05% of baseline values; *t*(60) = −10.534, *P* = 0.000 versus EA+NS group; and *n* = 10 per group, Figures 3(c) and 3(d)) compared to that in the EA+NS group (20–50 min after HFS: 207.48 ± 6.42% of baseline values; 50–80 min after HFS: 171.56 ± 8.16% of baseline values).

The analysis of dendritic spine density showed a significant difference among the control group, model group, and EA group (*F*(2,54) = 58.820, *P* = 0.000; *n* = 6 per group). The dendritic spine density was markedly decreased in model group rats (control rats: 11.29 ± 0.52, model rats: 6.21 ± 0.27, *P* < 0.01 versus the control group, Figure 3(f)), and it was significantly improved in EA group rats (EA rats: 9.21 ± 0.22, *P* < 0.01 versus the model group). In addition, the EA+H89 group rats had significantly fewer dendritic spines than those of the EA+NS group rats (5.20 ± 0.44 and 7.98 ± 0.67, resp.; *t*(19) = −3.821, *P* < 0.01 versus the EA+NS group, Figure 3(f)).

### 3.3. Significant Signs of Neuronal Loss Were Absent in Hippocampus in Experimental Rats from the Five Groups

Nissl stain was used to distinguish viable neurons from the apoptotic or the neurotic ones. The former exhibited abundant cytoplasm and Nissl substance (stained as dark blue), obvious oval nuclei (stained as light blue), and prominent nucleoli while the apoptotic or necrotic cells exhibited pyknotic morphology with amorphous or fragmented nuclei.

**Figure 4:**
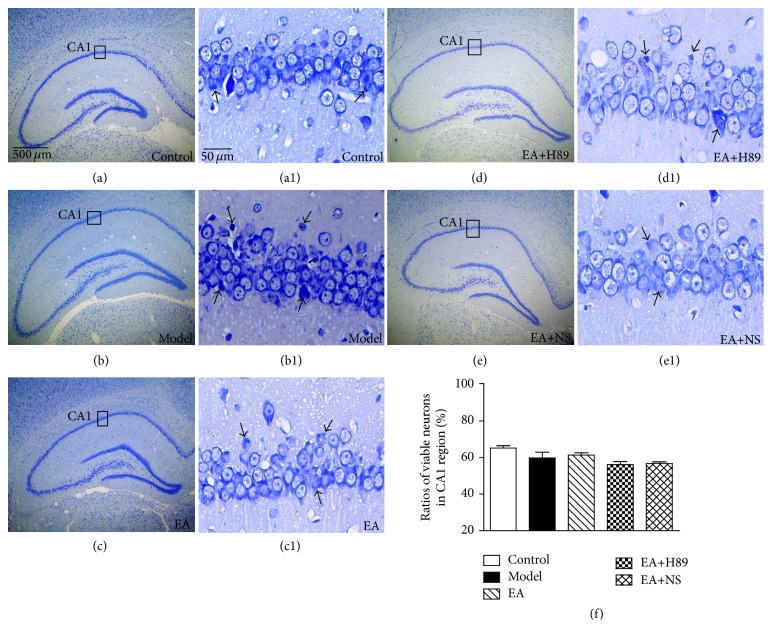
Significant signs of neuronal loss were absent in hippocampus in experimental rats from the five groups. Photomicrographs of toluidine blue-stained sections from control group (a and a1), model group (b and b1), EA group (c and c1), EA+H89 group (d and d1), and EA+NS group (e and e1). (a–e) 40x, scale bar = 500 *μ*m. (a1–e1) 400x, scale bar = 50 *μ*m. Significant signs of neuronal loss were absent based on the overall observation of hippocampus in experimental rats from the five groups. Several damaged neurons (black arrows) in CA1 pyramidal cell layer were observed. (f) Quantitative analysis of pyramidal cell viability in CA1 pyramidal cell layer was not significantly different neither among the control rats, the model rats, and the EA rats (control: 65.19 ± 1.25, model: 59.70 ± 3.20, and EA: 61.34 ± 1.15; *F*(2,109) = 3.075, *P* > 0.05; and *n* = 5 per group) nor between the EA+H89 rats and the EA+NS rats (EA+H89: 56.07 ± 1.77, EA+NS: 56.69 ± 0.93; *t*(26) = −0.336, *P* > 0.05; and *n* = 5 per group). Each value represents mean ± SEM.

Significant signs of neuronal loss were absent according to the overall observation in the hippocampus of experimental rats from the five groups (Figures 4(a)–4(e)). Considering previous reports that CA1 region is more vulnerable to ischemic damage [25], we focused on the neuronal damage in CA1 pyramidal cell layer. As shown in Figures 4(a1)–4(e1), several damaged neurons (black arrows) in CA1 pyramidal cell layer existed in the sections from the five groups. In Figure 4(f), quantitative and statistical analysis of pyramidal cell viability in CA1 pyramidal cell layer did not reach significance neither among control rats, model rats, and EA rats (control: 65.19 ± 1.25, model: 59.70 ± 3.20, and EA: 61.34 ± 1.15; *F*(2,109) = 3.075, *P* > 0.05; and *n* = 5 per group) nor between EA+H89 rats and EA+NS rats (EA+H89: 56.07 ± 1.77, EA+NS: 56.69 ± 0.93, *t*(26) = −0.336, *P* > 0.05; and *n* = 5 per group).

### 3.4. EA Increased the Expression of pCREB Protein, BDNF Protein, and miR132 and Decreased the Expression of p250GAP Protein in the Hippocampus of 2VO Rats and These Effects Were Partially Inhibited by H89

**Figure 5:**
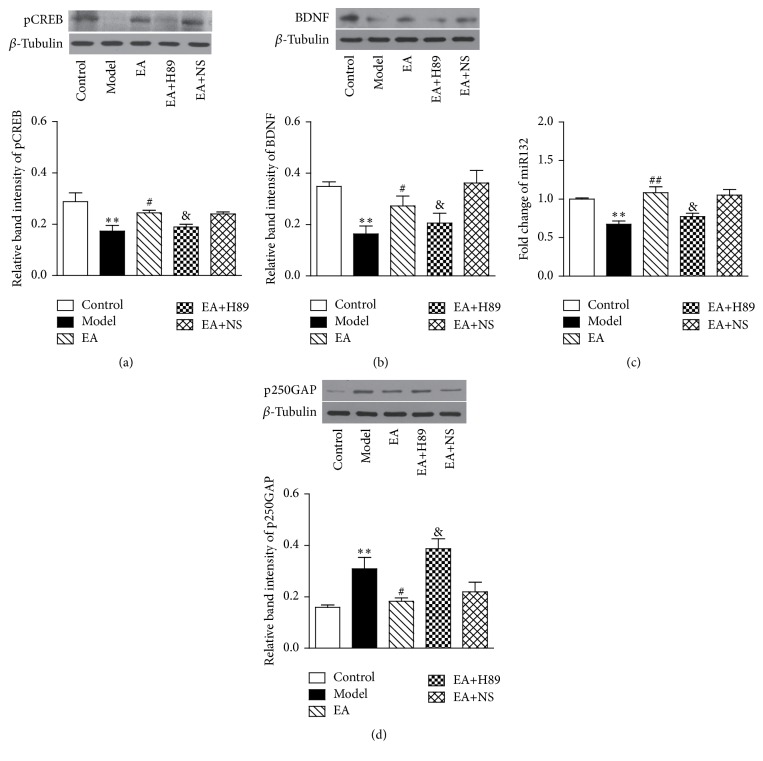
EA increased the expression of the pCREB protein, BDNF protein, and miR132 and decreased the expression of p250GAP protein in the hippocampus of 2VO rats and these effects were partially inhibited by H89. (a) Immunoblot analysis for pCREB protein in hippocampus and histogram of its relative band intensity. (b) Immunoblot analysis of BDNF protein in hippocampus and histogram of its relative band intensity. (c) Histogram of fold change of miR132 in the hippocampus. (d) Immunoblot analysis for p250GAP protein in hippocampus and histogram of its relative band intensity. Quantitative analysis demonstrated that hippocampal pCREB, BDNF, and miR132 levels were decreased by 2VO and increased by EA treatment. Concurrently, EA reduced the elevation of p250GAP level in the hippocampus of 2VO rats. These protective effects were at least partially inhibited by H89 administration. Each value represents mean ± SEM, *n* = 5 for each group. ^*∗∗*^ *P* < 0.01, compared with the control group; ^##^ *P* < 0.01, compared with the model group; ^#^ *P* < 0.05, compared with the model group; and ^&^ *P* < 0.05, compared with the EA+NS group.

Analyses with one-way ANOVA revealed a significant main effect of EA (pCREB: *F*(2,17) = 11.766, *P* < 0.01; BDNF: *F*(2,19) = 13.820, *P* = 0.000; miR132: *F*(2,33) = 9.106, *P* < 0.01; and p250GAP: *F*(2,17) = 10.539, *P* < 0.01; and *n* = 5 per group). As illustrated in Figure 5, the expressions of the pCREB protein, BDNF protein, and miR132 were decreased 7 days after the 2VO operation, accompanied by an increased expression of p250GAP protein (pCREB, BDNF, miR132, and p250GAP: *P* < 0.01, resp., versus the control group). Treatment of EA impeded the reduction of pCREB protein, BDNF protein, and miR132 expression in 2VO rats, as well as suppressing the increase of p250GAP protein (pCREB, BDNF, miR132, and p250GAP: *P* < 0.05, resp., versus the model group). Moreover, this protective effect of EA was partially reversed in EA+H89 group rats, when compared with the rats in EA+NS group (pCREB: *t*(4) = −3.290, *P* < 0.05; BDNF: *t*(7) = −2.711, *P* < 0.05; miR132: *t*(17) = −2.109, *P* < 0.05; and p250GAP: *t*(7) = 2.722, *P* < 0.05, versus the EA+NS group; and *n* = 5 per group, Figure 5).

## 4. Discussion

The present study has revealed that EA at GV20 and GV14 for 7 days could significantly ameliorate learning and memory deficits and LTP impairment in the Schaffer collateral pathway. In addition, it could also restore the dendritic spine loss in the hippocampal CA1 region in cerebral hypoperfusion rats. PKA/CREB signaling pathway was potentially involved in the neuroprotective effects, including the regulation of proteins such as pCREB, BDNF, p250GAP, and miR132.

The 2VO rat model is widely used to study the chronic cerebral hypoperfusion- (CCH-) related cognitive impairment. The subchronic phase of this model was defined as 3 days–8 weeks after the occlusion, causing a dramatic reduction in cerebral blood flow (CBF) and hypoxic-ischemic conditions [26]. Even if the CBF gradually returns to the baseline with the compensatory mechanism of vascular plasticity, such cerebral hypoperfusion may be a risk factor for subsequent neuronal degeneration and cognitive impairment [27]. In Schaffer-CA1 synapses, the LTP was significantly inhibited at both 1 day and 4 days after clamping of the bilateral common carotid arteries; that is, a so-called delayed dysfunction might exist in the hippocampal neurons [28]. Consecutively, it was demonstrated that the synaptic transmission reduction of the hippocampus and prefrontal cortex was partially attributed to the dendritic spines loss in the hippocampus, which contributed to the learning and memory dysfunctions in 2VO rats [29]. Therefore, interventions that reverse, at least partly, early neuropathological outcomes of CCH might be an effective way to prevent the progressive decline of cognitive functions. In this study, EA could significantly reverse the LTP impairment and dendritic spine loss, indicating a beneficial role in improving the neuronal synaptic transmission and functional reconstruction in cerebral hypoperfusion rats.

Significant pathological changes in the brain may be absent at early stages of bilateral common carotid ligation. A previous study had shown that the pyramidal cell loss in the unilateral hippocampal CA1 region was only observed in a few 2VO rats (1/6) on day 7 after the surgical procedure [30]. Similarly, it was reported that 2VO did not influence the hippocampal CA1 pyramidal cell number or density of glial fibrillary acidic protein (GFAP) 14 days after surgery, though a late-emerging CA1 cell loss was detected at 190 days after the operation [31]. Together, these findings have prompted a consensus that initial 2VO does not cause significant neuronal loss. Consistent with these reports, our experimental outcomes also detected minimal differences in viable hippocampal neurons among the experimental rats.

CREB is best known for its roles in learning and memory. Over the past few decades, accumulated evidence established that pCREB-mediated gene transcription and protein synthesis contributed to the development of long-term memory [32]. This was brought about by participating in processes such as long-term potentiation of synaptic strength [15, 32] and structural synaptic changes. In a gerbil global ischemia model, preconditioning ischemia induced ischemic tolerance by a transient increase of pCREB and subsequent upregulation of BCL-2 expression [33]. However, ongoing chronic ischemia reduced the expression of pCREB, resulting in the selective vulnerability of CA1 pyramidal cells at 48 and 72 h following mild hypoxic-ischemic (HI) injury in rats [25]. In the present study, we found that EA could increase the expression of the pCREB protein in the hippocampus of 2VO rats, suggesting that EA might improve learning and memory through activation of CREB mediating gene transcription and protein synthesis.

Sufficient evidences have suggested that CREB is a major regulator of BDNF-induced neuronal responses. On one hand, the transcription and translation of BDNF gene are CREB-dependent [34, 35]; on the other hand, BDNF could stimulate Ser133 phosphorylation and activate CREB through CaMKIV [36] and RSKs [37]. Thus, a positive feedback loop might have connected BDNF and CREB. Meanwhile, BDNF was demonstrated to play a crucial role in synaptogenesis [38] and LTP [39, 40]. Moreover, it was reported to enhance high-frequency transmission [41] and mediate the redistribution of the synaptic proteins within the presynaptic terminals. Similar findings were observed in this study: that hippocampal BDNF protein expression was decreased after the 2VO procedure and increased after the EA treatment, accompanied by alleviation of LTP impairment and partial restoration of dendritic spine density.

We also observed another newly recognized CREB-driven target, miR132, which was indicated in mediating dendritic plasticity through suppressing translation of p250GAP, a member of the Rac/Rho family of GAPs [21, 42]. As reported, the transcription of miR132 was induced in response to neurotrophins [43] or synaptic activity [44]. BDNF could regulate miR132 transcription via the ERK1/2 signaling, together with kinase MSK1 and the phosphorylation of CREB [43]. Herein, we found that EA altered the morphology of dendritic spines, accompanied by an increase of miRNA-132 and a reduction of p250GAP protein in 2VO rats. These results revealed the important role of miR132 in the improvement of learning and memory by EA.

A group of rats were pregiven H89 [45] through icv injection and then received operation of 2VO and EA treatments. Interesting results were found that H89 administration could reverse the beneficial effects of EA on spatial learning and memory, including LTP facilitation and restoration of dendritic spines. Simultaneously, the effects of EA on the molecular level of pCREB protein, BDNF protein, miR132, and p250GAP protein were also blocked by H89 in EA-treated 2VO rats. All these results implied a potential involvement of PKA/CREB signal pathway in the effects of EA on cognitive function and hippocampal synaptic plasticity in cerebral hypoperfusion.

## 5. Conclusions

Taken together, our data indicated that EA could ameliorate learning and memory deficits and alleviate hippocampal synaptic plasticity impairment of cerebral hypoperfusion rats, with a probable regulation of PKA/CREB signaling pathway, including the expression of pCREB protein, BDNF protein, miR132, and p250GAP protein.
